# Assessment of Maya women’s knowledge, attitudes, and beliefs on sexually transmitted infections in Guatemala: a qualitative pilot study

**DOI:** 10.1186/s12905-020-00925-7

**Published:** 2020-03-21

**Authors:** Noor Tasnim, Emma M. Heneine, Casey M. MacDermod, Maria L. Perez, David L. Boyd

**Affiliations:** Duke Global Health Institute, Duke University, 310 Trent Dr, Durham, NC 27710 USA

**Keywords:** Guatemala, Maya, Women, Health, Sex, STIs, Knowledge, Attitude, Beliefs, Prevention

## Abstract

**Background:**

Despite Guatemala’s large indigenous population, indigenous health is often neglected in reported health data and interventions. Although this data is limited in scope, it shows that indigenous people have poorer health outcomes. Sexually transmitted infections (STIs) are now a growing threat in Guatemala and pose great risk to the wellbeing of its indigenous population.

**Methods:**

This qualitative pilot study assessed the knowledge, attitudes, and beliefs of STIs through semi-structured interviews among a previously unstudied population of indigenous Maya women (*n* = 35, ages 18–50) in the six municipalities of Santa Cruz La Laguna, Guatemala.

**Results:**

Four key themes were identified: 1) indigenous Maya women have limited factual knowledge about sex and STIs; 2) widespread partner infidelity minimizes women’s control over preventing STI contraction; 3) close-knit communities and the resulting heightened fear of gossip prevents communication and hinders care seeking; and 4) lack of quality medical care and inaccessibility of biomedical healthcare systems pose barriers to seeking care for potential STIs.

**Conclusions:**

To address these findings, we suggest methods to improve sexual education, combat male infidelity, promote condom use, and improve health services to reduce the incidence of STIs in Maya Guatemala.

## Background

### Indigenous health in Guatemala

The health outcomes of indigenous populations around the world are dramatically worse than those of other groups living in the same country [1–3]. Factors such as discrimination, poverty, and lack of education and health care exacerbate this inequity [4]. Language and location barriers historically have inhibited data collection and have left indigenous populations’ health outcomes unexamined [2, 4, 5]. There are currently 370 million indigenous people living in 90 countries. It is now a global health priority to address their health disparities and increase their quality of life [3].

Guatemala is home to the largest number of indigenous people in Central America [3]. Approximately 38.5% of the nation’s population is Mayan [6]. Most of the Maya live in the rural areas of the Central, North, Northwest, and Northeast regions of Guatemala and many speak little to no Spanish but rather one of the many Mayan languages [6, 7]. They have limited access to health care and education and generally have worse reported health outcomes compared to Guatemala’s non-indigenous population [6, 7]. Guatemala’s national surveys comprehensively collect data on the health of its population; however, stigma surrounding sex poses an obstacle to properly assess sexual health in the nation [8, 9].

### Sexual health in Guatemala

The incidence of sexually transmitted infections (STIs) continues to remain high throughout the world [10]. In Guatemala, deaths associated with human immunodeficiency virus (HIV) and acquired immunodeficiency syndrome (AIDS) increased by 167 and 23%, respectively, between 2010 and 2016 [11]. In 2016, only 19% of Guatemala’s 46,000 HIV positive individuals had antiretroviral therapy [11]. In the same year, only 19% of pregnant women with HIV received treatment/prophylaxis to prevent HIV transmission during childbirth [11]. The risk of STIs, particularly HIV/AIDS, is a growing threat to Guatemalans’ well-being and must be addressed immediately.

The increase in reported STIs is linked to the high levels of migratory work in Guatemala. Men living around Lake Atitlán in the Mayan Western Highlands, in particular, are often migrant workers, travelling far from home to find work to support their families [12]. Women whose partners are migrant workers are twice as likely to report STI symptoms than women whose partners do not travel for work [12]. This phenomenon may be explained by men’s tendency to seek services from female sex workers (FSWs) while away from home. In fact, Guatemalan FSWs have a higher prevalence of STIs than the general population [9, 13–15]. Men’s risk of contracting STIs is exacerbated by the high levels of unprotected sex with FSWs [9]. To address STI prevalence among FSWs, the Guatemalan government now requires them to certify their routine visits to health clinics [9, 14, 15]. However, there are no laws regulating how men seek FSWs’ services or test for personal STIs [9]. Thus, the existing regulation is insufficient and does not eliminate the risk of STI transmission between FSWs and men.

Rigid gender roles further contribute to STI transmission. The masculinity norms associated with “machismo” culture can lead Guatemalan men to feel invincible in relationships [16]. This invulnerability, in turn, discourages men from receiving necessary check-ups at health clinics [16]. Moreover, it can result in men’s sexual entitlement [13]. As a result, men may have multiple sexual partners and infrequently use condoms [13]. Poor health seeking behaviors, paired with low condom use with multiple sexual partners, including FSWs, increase men’s risk of contracting STIs [13]. The risk of contracting STIs is not limited to men, however. As infected men return to their spouses or other sexual partners, they leave their partners susceptible to STI contraction. In fact, women in developing nations, like Guatemala, have suggested that men serve as “the bridge” of STI transmission between FSWs and the greater population of women [12, 13, 15]. Women, therefore, are particularly vulnerable to STI contraction, as much of their partners’ actions remain outside of their control.

Stigma surrounding sex, especially STIs, poses an additional obstacle to promoting sexual health in Guatemala. Rather than going to health clinics to treat their STIs, male clientele of FSWs often rely on self-medication, traditional medicine, and other confidential options because of shame [9]. FSWs have reported dissatisfaction with health clinics because of the perceived negative judgement they receive, associated with STIs and sex work [8]. Because of the stigma surrounding STIs, and sex in general, both men and women are discouraged from visiting clinics to test for and/or treat potential STIs. This exacerbates the spread of STIs in Guatemala and lowers the number of reported STIs, thus yielding inaccurate statistics on STI prevalence and incidence.

### Indigenous sexual health in Guatemala

There is a paucity of studies on STIs within indigenous populations [14, 15]. Most studies about indigenous peoples have focused on populations in developed nations [17]. Available data show that STIs are increasing among indigenous populations and the knowledge gap on their rates needs to be addressed [17]. In Guatemala, there only exists literature examining indigenous adolescents’ perceptions of STIs in the Western Highlands [18]. Most studies on STIs in Latin America, including Guatemala, have focused on STI prevalence primarily within FSW populations [14, 15].

No study has assessed a broad age range of indigenous Maya women’s perspectives on STIs. Because of the large concentration of indigenous communities in the area, Lake Atitlán in Guatemala’s Western Highlands is an optimal site for assessing these perceptions. Men in the region are also migrant workers, increasing their risk of encountering FSWs, contracting STIs, and transmitting them to their partners. To better understand and promote indigenous populations’ sexual health, this study assessed Maya women’s knowledge, attitudes, and beliefs (KAB) of STIs in the Lake Atitlán region. These results can guide interventions to empower indigenous women in rural Guatemala through protecting and improving their sexual and reproductive health.

## Methods

**Fig. 1:**
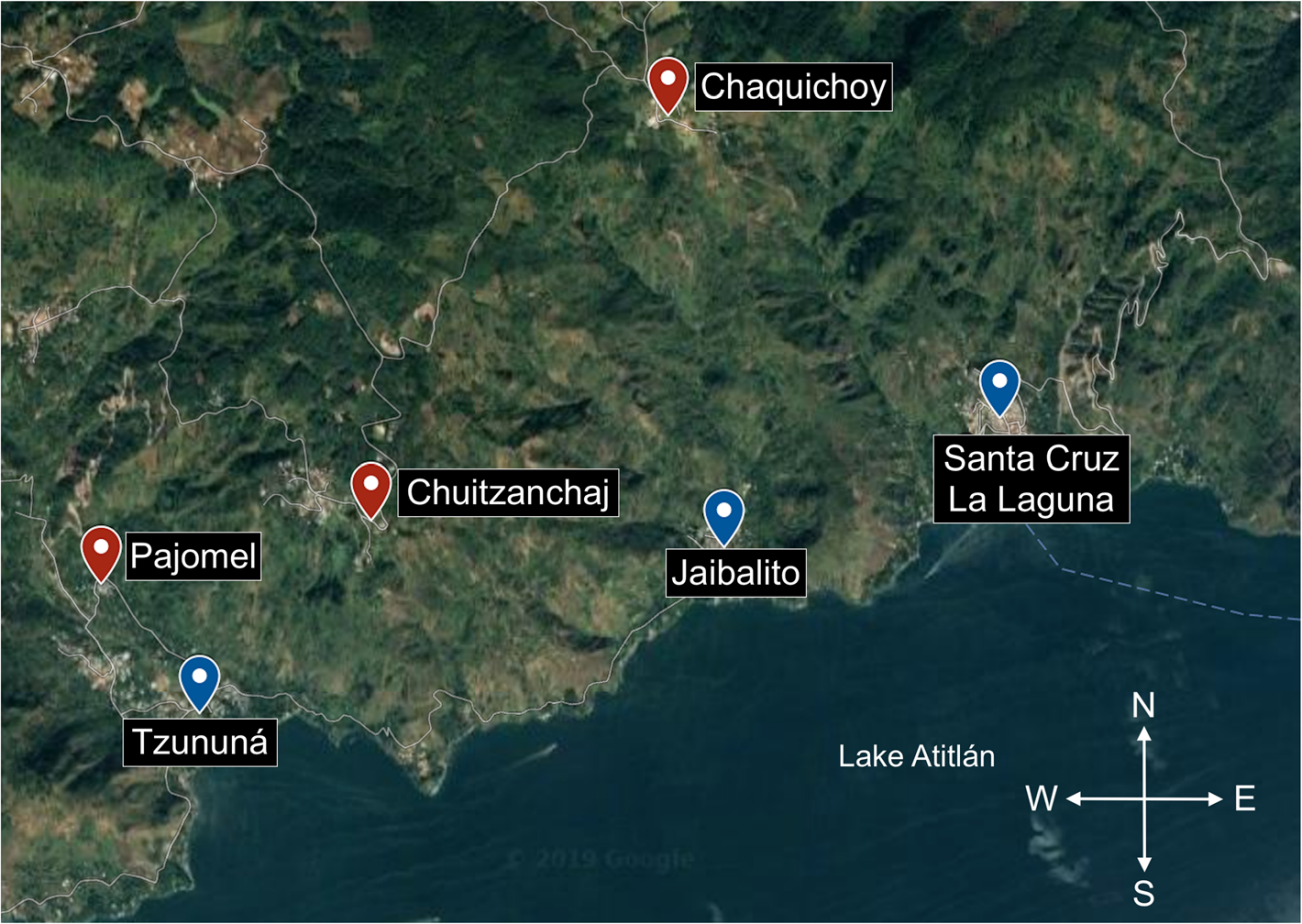
Locations of the villages surrounding Lake Atitlán*.* “Lower villages” are in blue, “upper villages” are in red. Map of Guatemala rendered with Google Maps [19]

The pilot study employed qualitative measures in six neighboring villages in the municipality of Santa Cruz La Laguna, located near the shore of Lake Atitlán in the Western Highlands of Guatemala: Santa Cruz, Tzununá, Jaibalito, Chaquijchoy, Chuitzanchaj, and Pajomel (Fig. 1). This location was chosen because of the large population of Mayans that live in the area. Moreover, the municipality’s accessibility to Lake Atitlán is the reason why it was chosen above other locations in Guatemala. This proximity promotes migratory work that can increase susceptibility to STIs for communities in the region [12]. The more populous and accessible lower villages located on the lakefront (Santa Cruz, Tzununá, and Jaibalito) use the lake as their primary entry point and house the only two health clinics in the municipality. By contrast, the less populated, more impoverished, and isolated upper villages, (Chaquijchoy, Chuitzanchaj, and Pajomel) located in the mountains above the lake, are accessible only by a partially paved mountain road [20].

Using clustered convenience sampling, our collaborators – two local women fluent in Spanish and Kaqchikel with over a decade of experience in public health – identified 35 female participants. The participants of this pilot study were a small portion of the nearly 3000 women that live in Santa Cruz La Laguna [20]. Participants aged 18–50 years (average age = 34 years) from about 6 households per village represented a range of demographic characteristics broadly representative of women in the region (Additional file 1). We assume there were menopausal women in this sample as some participants were near the age of 50. Although we did not collect this data directly, one of our oldest participants reported that her doctor told her to stop taking contraceptives because she had gone through menopause. To participate, women gave informed consent to be interviewed and audio recorded. The study was approved by the Institutional Review Board Committee of Duke University.

After a comprehensive literature review, semi-structured interview questions related to KAB about sex and STIs were developed. Questions were first translated from English to Spanish, then reviewed, translated, and backtranslated into a culturally appropriate Kaqchikel script by the collaborators who also served as interpreters during interviews (Additional files 2 and 3).

Interviews were conducted in participants’ homes between June and August 2016. During interviews, one researcher recorded responses while the other asked questions in Spanish. The interpreter translated questions and participants’ responses (Spanish-Kaqchikel). The few participants who spoke Spanish were interviewed directly by a researcher in Spanish. Four researchers transcribed the Spanish portions of the audio recordings. The framework method was used to categorize major themes in responses [21]. Each researcher read through and coded the same subset of transcripts (*n* = 9; 25%) until inter-coder reliability was reached and saturation was met. The remaining transcripts were single coded.

## Results

### Limited knowledge on STIs and sex

The first theme is that indigenous Maya women have limited factual knowledge on STIs and sex. In fact, most women received little to no sexual education before marriage. Nearly half of the participants reported not having received any information about sex before marriage (*n* = 17).

*“She says that she has received a little information about sex in capacitaciones,*^***1***^*nothing more because her family never talked about it. Her daughters also talk about the subject with her at times because they are studying it away from the community and then discuss what they learn with her.”*

*“She never had information before marriage. Nothing. Not from her mother, aunts, nor female friends. She never thought about how to have sex. Nothing, nothing until she married her husband.”*

Sources of sexual education in the lower villages were more diverse than those of upper villages. Within the lower villages, the most frequently reported sources of sexual education were mothers (*n* = 5), capacitaciones^1^ (*n* = 4), and health clinics (*n* = 2). In the upper villages, only capacitaciones (*n* = 6), mothers (*n* = 1), and television (n = 1) were mentioned. Types of information that women reported receiving included family creation/pregnancy/motherhood (*n* = 5); menstruation (*n* = 3); condoms/contraceptives (*n* = 3); sex (*n* = 2), and STIs (*n* = 1). Only one of the 13 participants who was asked about their first menstruation had prior knowledge of puberty and menstruation. The remaining 12 women reported feeling shocked or scared, with several feeling too ashamed or embarrassed to ask someone about their menses.

*“When she was 7 years old, her mother passed away and they were four [sisters] and each one cried when they first menstruated because there was no one there to speak with them [about it], nor had someone talked to them about it prior. Because this happened, some female neighbors began to speak with them and told them not to worry – that this is normal for a woman and begins when someone reaches this age, hence, it’s normal. Since then, they understood that it is normal.”*

*“When she was little, her mother passed away and no one warned her [about menstruation] until she had [her first period]. She was scared because she thought it could be an illness or something else. Hence, she thought she would die. ‘I’m dying, I’m dying,’ she said. She had the same reaction when days passed and the other month came. Afterwards, she was encouraged to tell some of her female friends who knew and told her not to worry, this happens every month and is normal for women. Since then, she has understood that [menstruation] is normal.”*

Our respondents also had limited factual knowledge about STI symptoms. Among the 10 participants who could name an STI, only AIDS was mentioned. Roughly 43% of respondents (*n* = 15) could not name any STI symptoms. The 20 remaining participants mentioned a range of STI symptoms, with only one woman acknowledging that symptoms differed per illness. Among the most commonly mentioned symptoms were death (*n* = 10), weakness/debilitation/inability to work (*n* = 10), and losing appetite and weight (*n* = 5). One woman noted that HIV/AIDS positive women cannot breastfeed. Itchiness, fever, and deterioration of the body/genitals were each mentioned twice by different participants.

Participants were asked how to prevent STIs. Fidelity was the most common response, followed by condoms and going to the doctor. Nearly a quarter of respondents reported that they did not know. The majority of women (*n* = 30, 85%) knew what condoms were; however, only 6 (lower village = 5, upper village =1) women knew how to use them.

### Partner infidelity minimizes women’s control over STI prevention

The second theme identified is that widespread partner infidelity minimizes women’s control over preventing STI contraction. During the course of the interviews, nearly all of the women (*n* = 33, 94%) explicitly linked STIs with infidelity in some way, with 24 (68%) specifically linking STIs with infidelity in men. Among the women who had knowledge about STIs, 75% (*n* = 24) mentioned infidelity when asked about STI transmission and contraction, with 13 specifically mentioning that STIs are passed from unfaithful husbands.

*“[STI transmission] depends on whether men or women leave their town. They find people not from their town, for example, who sometimes come to Panajachel. From there, they bring the illness. She said there is this countryman that went to the States and contracted this illness. When he came back, he had sex with other women and infected them.”*

*“Men are going to look for other women in places close to the town or here as well. And there are women like this who have contracted sexually transmitted infections … This is bad because [these men] infect other people or their partner. Not all of them notify if they have an illness. They hide it and endure it.”*

Women reported wide prevalence of male infidelity. We asked participants who they believe had more STIs, men or women. Of the 30 respondents, 20 participants (67%) believed men have more STIs. The most frequently reported reason for this was men’s freedom to travel outside the community, often for work (*n* = 10). We also asked 33 women if they thought married men in their communities had “lovers” – partners outside of their committed relationships. A majority of these women (*n* = 26) claimed that men in their communities had lovers. Four of the five women who believed not many unfaithful men existed in their community were from one of the upper villages.

*“Yes [I believe married men in my community have lovers] because my husband did the same. I am in my second marriage. I already had my first marriage.”*

Women have limited control over their husband’s condom use, as they are commonly associated with infidelity. The 30 women who reported knowing what condoms are were asked a series of questions about condom use. Most of these women believed that members of their communities used condoms and a handful believed that women in their communities asked men to wear condoms. When asked if requesting men to use condoms would make them angry, most of these women reported that it depended on the situation. Of the 10 women who specified if men use condoms with lovers or with their wives, 9 respondents said that men use condoms exclusively or almost exclusively with lovers.

*“She once went to the field to look for firewood and found a thrown away [condom]. A friend of hers said this object was used by men who have lovers”*

### Fear of gossip hinders communication and care seeking

The third theme is that close-knit communities and the resulting heightened fear of gossip prevents communication and hinders care seeking. Knowledge of community members’ lives was found to be commonplace. Thus, women frequently reported shame, embarrassment, and fear of gossip as deterrents to talking about STIs and sex in their communities. Although the majority of respondents said they have already advised their children or plan to do so on sexual matters (*n* = 29, 91%), 2 respondents (6%) from the lower villages were confident that they will not speak to their kids about such matters due to shame and embarrassment. Women reported talking to their children on general life advice, not sexual relations specifically. Common topics of advice included postponing marriage/parenthood (*n* = 5), telling daughters to be careful with men (*n* = 5), and to “cuidarse”^2^ from STIs and pregnancy (*n* = 5).

*“At the moment, she has not spoken to her children [about sexual relations]. When they are older, she wants to talk to them so that they do not suffer … She would advise them on what is good and bad so that they understand how life is … She fears for her children because young boys are leaving girls pregnant... She does not want this to happen to her children and has to advise them to do good things so that they are careful.”*

We asked participants if they would tell either their children, friends, partner, and/or doctor about a personal STI. None of the participants said they would tell their children. Only 2 women said they would tell their friends in order to warn them and 4 respondents said it depended on their trust or the severity of their illness. The remaining 26 respondents (81%) said they would not tell their friends about a personal STI, most often because of shame (*n* = 12) or fear (*n* = 8). Women (upper villages = 5, lower villages = 1) reported fear of gossip as a deterrent to telling their friends about personal illness. 22 participants said they would not tell their partners about their personal STI either. The leading reason for not telling one’s partner about personal illness was fear of violence, separation and/or other consequences (*n* = 16). Additionally, 7 participants said it depended on the situation, with 2 claiming they would tell their husbands if they themselves had been faithful. Ultimately, only 2 women said they would tell their partners about a personal STI, both wanting to address and preserve everyone’s health. One woman expressed concern that she had an STI but said she would not tell her husband about it out of fear.

*“No [she would not tell her partner about a personal disease] so that he does not hit her or leave her. Therefore, she would have to endure this.”*

*“She is infected, and her genitals are in a lot of pain, but she does not have economic resources and cannot [go to the doctor]. She has not told her husband because he would think that she has another partner.”*

The majority of women we interviewed believed there was at least some STI prevalence in their communities. Not having heard talk about STIs was the predominant reason given from respondents who didn’t believe STIs existed in their respective communities in the upper villages.

### Lack of quality healthcare

The final theme is that lack of quality medical care and inaccessibility of biomedical healthcare systems pose barriers to seeking care for potential STIs. Participants reported a general willingness to visit the doctor for treatment of STI-related symptoms. However, stigma and bad experiences with biomedical healthcare were found to notably inhibit care seeking. We asked participants what they would do if they experienced a series of symptoms commonly associated with STI contraction (pain while urinating, abnormal discharge, uterine pain, genital itching, and abnormal vaginal bleeding), to gauge their preferred treatment regimen. Nearly all of the respondents (*n* = 31, 88%) would go to the doctor for one or more of the aforementioned STI symptoms, with 4 women saying they wouldn’t go to the doctor, 1 of whom clarified that it was because she didn’t have any money.

Although not a question on our survey, 9 women shared that they personally had a bad experience with the biomedical healthcare system. Additionally, 9 women reported significant economic barriers prevented them from seeking care, and 11 mentioned that they would have to travel outside of their community to seek care (4 from the lower villages, 5 from the upper villages). Several women offered stories of past illnesses and their healthcare seeking behavior. Poor quality, inaccessible, and expensive biomedical care, in turn, led our participants to rely on traditional medicine, such as the temazcal^3^ and herbs, to treat STI-related symptoms.

*“Her husband won’t give her money to see a doctor so she sold her animals and went to Santa Cruz to see a healer, but that also costs money and she has suffered for ten years”*

*“Throughout history, this area’s doctors have sometimes not explained what someone has. They only say what you need to take. This is what they did to her. They did not tell her what she had but gave her vaginal ovules. She used them, and from there, she was cured.”*

Women were also asked questions related to trust and comfort with doctors. When asked if they would tell their doctor if they had an STI, 20 of the 25 respondents (67%) said they would, with shame preventing 3 women who said they would not tell a doctor about a personal STI. Relatedly, half of the respondents who were asked about their preference for a male or female doctor claimed they had no preference (*n* = 9, 50%). The 3 participants who preferred male doctors all claimed that they know more male than female doctors. The remaining 7 respondents preferred female doctors because they trust them more (*n* = 3) and do not feel ashamed with them (*n* = 2).

## Discussion

Several factors in combination can help explain the themes identified in women’s KAB about STIs. Financial barriers to schooling, inadequate sexual education, and stigma contributed to limited factual knowledge about STIs and sex in educational and non-educational spaces alike. Early marriages, paired with men’s migratory work and low condom use, minimized women’s control over STI contraction as a result of their partners’ widespread infidelity. Moreover, the small sizes of our participants’ communities, gossip, and stigma associated with sex and STIs prevented communication and care-seeking behavior. Lastly, negative experiences with biomedical healthcare, costs, and the lack of access to healthcare deterred our participants from seeking care for potential STIs. These themes and factors explain why there is such a high risk of contracting STIs for Maya women in this region.

Our study is the first to assess KAB of indigenous Maya women on sex and STIs. Our results expand on previous literature on STIs and associated risk while applying similar findings to a population that is rarely studied. Indigenous populations historically have reported worse health outcomes as a result of poverty and limited access to health care and education [1–4]. These factors have prevented Maya women from attaining the care and education they need to prevent STI contraction and transmission. Additionally, stigma has become a large inhibitor of health-seeking behavior in Guatemala [8, 9]. Our results show that this especially applies to Maya women, who live in small communities that allow stigma to prevent communication between them and their partners, family members, friends, and healthcare providers. Evidence of “machismo” culture prevailed in our results as well. Echoing findings in similar studies, many of our participants reported that their partners and men in their communities exhibited sexual entitlement while traveling as migratory workers and having sex with other women, perhaps FSWs [13, 16]. These studies also reported low condom use by men as a result of this entitlement [13, 16]. This was observed in our study and similarly prevented Maya women from having control over STI contraction from their male partners.

There were a few limitations to our study. First, the extensive nature of our interviews limited our sample size; however, we were still able to collect robust data from the women that participated in our study. The sample size was limited because our study focused on a topic typically associated with stigma and seldom discussed within local communities. As a result, we encountered women who were not willing to participate in our study. Moreover, the less populous upper villages provided us with small pools from which we could sample. In an attempt to ensure equal representation of all the villages in our study, a limited number of women were recruited. A language barrier also existed when recording our responses. Because the researchers were not fluent in Kaqchikel, participants’ responses had to be translated to Spanish by our local collaborators so that they could be recorded. As a result, some participants’ responses, especially those that were longer, may have been paraphrased by our collaborators, preventing us from collecting more thorough data. Nevertheless, this study is an important first step towards addressing previous approaches that have noted indigenous languages as “implementation barriers” or have ignored indigenous populations in global health work [2]. Lastly, it is possible that some of our participants were reserved in their responses and were not comfortable sharing all the details associated with their experiences regarding STIs and sex. Still, our participants provided us with extremely valuable data that can guide future interventions.

## Conclusion

This research has revealed critical insights into Guatemalan Maya women’s KAB surrounding STIs. Data gathered from the interviews, paired with recent surges in reported STIs, demonstrate that women’s sexual and reproductive health needs are not being met. Our analyses suggest that this is, in large part, due to insufficient sexual education, stigma around sex, widespread male infidelity, minimal condom use, and inefficient health services and that interventions should be designed to address these findings and ultimately promote the sexual and reproductive health of indigenous Maya women living around Lake Atitlán. Coupled with further research, recommendations such as these can serve as a starting point for developing a fully comprehensive, culturally relevant approach to improving indigenous women’s sexual and reproductive health.

### Strengthening sexual education

We recommend that sexual education is strengthened in schools and capacitaciones. In primary and secondary schools, youth, especially girls, are a particularly vulnerable subpopulation. With frequent teenage marriages and pregnancies, basic sexual education is essential for puberty-aged kids. Capacitaciones were also found to be a principle source of sexual education for women. As an established, reputable, and trusted source, capacitaciones are an important and underused resource for disseminating critical sexual education.

### Combating male infidelity and “machismo”

Targeted interventions and programs designed to promote gender equality through the perspective of boys and men are effective and underused methods to support women and girls. Involving boys and men is necessary to transform gender norms, challenge patriarchal control, and empower girls and women. Moreover, continued education should be encouraged within these communities. More years of education, especially among girls, is associated with delayed marriage. Prolonging the time for sexual and romantic exploration, as well as personal development, before marriage is necessary to combat the high levels of infidelity resulting from early marriages.

### Promoting condom use

Overall, condom use was low, especially among married couples. Condoms were found to be associated with infidelity. Thus, increased marketing of condoms to men and women as a way to prevent both pregnancy and STI transmission is needed to improve community perceptions of condoms and promote their use. Additionally, many women reported feeling too embarrassed or ashamed to buy condoms. Increasing the supply of condoms in health clinics, pharmacies, and community shops can help normalize their existence and combat the stigma associated with sex and condoms. Making condoms readily available can promote condom use among sexual partners, including married couples.

### Improving health services

There is a need for greater patient-provider transparency. Previous physicians’ failure to disclose information on Maya women’s health status has notably discouraged careseeking among Maya women. Improved physician training and increasing provider-patient communication is necessary to encourage careseeking and establishing trust with communities. We also recommend increasing the number of women in health services. Several women noted a preference for female doctors whom they trust more and feel less ashamed with than their male counterparts. Ensuring all subpopulations’ comfort and trust is crucial to promoting careseeking.

## Supplementary information

**Additional file 1.** Demographic Information of Participants

**Additional file 2.** Interview Questions in Spanish

**Additional file 3.** Interview Questions in English

## Data Availability

The datasets used and/or analyzed during the current study are available from the corresponding author on reasonable request.

## Abbreviations

STIs: Sexually transmitted infections
HIV: Human immunodeficiency virus
AIDS: Acquired immunodeficiency syndrome
FSWs: Female sex workers
KABs: Knowledge, attitudes, and beliefs
CONALFA: Comite Nacional de Alfabetización

## Footnotes

1 Capacitaciones are educational workshops and trainings open to the public, led by local organizations.

2 Translates to “take care [of yourself]” or “be careful.” When asked to elaborate on what was meant by that, women mentioned staying faithful to partners and not having sexual relations with others.

3 A wood-fired sauna used by the Maya for bathing and healing.
