# Supplementary material for: Assessment of Maya women’s knowledge, attitudes, and beliefs on sexually transmitted infections in Guatemala: a qualitative pilot study
Source: BMC Womens Health. 2020 Mar 21;20:58. doi: 10.1186/s12905-020-00925-7 (PMC7085160; doi:10.1186/s12905-020-00925-7)
Supplement: Supplementary file 1 — Additional file 1. Demographic Information of Participants [file 12905_2020_925_MOESM1_ESM.docx]

**Additional File 1. Demographic Information of Participants**

| **Age Range at Study Participation** | **Have they received schooling?** | **Current Number of Children** | **Marital Status** | **Age Range at Marriage** |
| --- | --- | --- | --- | --- |
| 21-25 | No | 2 | Married | 15-20 |
| 26-30 | No | 1 (adopted) | Single | NA |
| 41-45 | No | 1 (3 have died) | Married | 15-20 |
| 46-50 | Yes (until 1^st^ Grade) | 7 | Married | 15-20 |
| 46-50 | No | 5 | Married | 15-20 |
| 21-25 | No | 2 | Married | 15-20 |
| 46-50 | Yes (for 6 months) | 6 | Married | 15-20 |
| 18-20 | Yes (until 4^th^ Grade) | 2 | Unmarried Partner | NA |
| 21-25 | Yes (until 6^th^ Grade) | 1 | Unmarried Partner | NA |
| 26-30 | Yes (until 2^nd^ Grade) | 3 | Married | 15-20 |
| 36-40 | No | 4 | NA | NA |
| 36-40 | No | 3 | Married | 15-20 |
| 36-40 | No | 6 | Married | 15-20 |
| 26-30 | No | 2 | Married | 21-25 |
| 36-40 | Yes (until 1^st^ Grade) | 2 | Married | Under 15 |
| 26-30 | Yes (until 6^th^ Grade) | 2 | Married | 21-25 |
| 26-30 | Yes (until 1^st^ Grade) | 2 | Married | 15-20 |
| 31-35 | No | 6 | Married | 15-20 |
| 46-50 | Yes (until 1^st^ Grade) | 7 | Married | 15-20 |
| 41-45 | No | 6 | Married | 15-20 |
| 46-50 | No | 8 | Married | 15-20 |
| 41-45 | No | 6 | Separated | Under 15 |
| 41-45 | No | 6 | Married | 15-20 |
| 26-30 | Yes (for 2 years) | 4 | Married | 15-20 |
| 21-25 | No | 0 | Single | NA |
| 21-25 | Yes (until 6^th^ Grade) | 1 | Married | 15-20 |
| 41-45 | No | 10 (2 have died) | Married | 15-20 |
| 21-25 | Yes (until 6^th^ Grade) | 1 | Married | 15-20 |
| 26-30 | No | 3 | Married | 15-20 |
| 26-30 | Yes (for 2 years) | 4 | Married | 15-20 |
| 36-40 | Yes (for 6 months) | 5 | Married | 15-20 |
| 26-30 | Yes (for 3 years) | 5 | Married | 15-20 |
| 41-45 | No | 5 (1 has died) | Married | 15-20 |
| 21-25 | Yes (for 3 years) | 3 | Married | 15-20 |
| 21-25 | Yes (until 6^th^ Grade) | 1 | Married | 15-20 |
