# Supplementary material for: Assessment of Maya women’s knowledge, attitudes, and beliefs on sexually transmitted infections in Guatemala: a qualitative pilot study
Source: BMC Womens Health. 2020 Mar 21;20:58. doi: 10.1186/s12905-020-00925-7 (PMC7085160; doi:10.1186/s12905-020-00925-7)
Supplement: Supplementary file 2 — Additional file 2. Interview Questions in Spanish [file 12905_2020_925_MOESM2_ESM.docx]

**Additional File 2. Interview Questions in Spanish**

*Información demográfica*

1. ¿En qué sector vive?

2. ¿Cómo se llama? ¿y su apellido?

3. ¿Cuántos años tiene?

4. ¿Ha asistido a la escuela? ¿Por cuántos años?

5. ¿Cuántos hijos tiene?

6. ¿Cuántas personas viven en su casa? ¿Quiénes son?

7. ¿Usted tiene un esposo, una pareja, un marido, o un novio?

8. ¿Cuántos años tenía cuando usted se casó?

9. ¿Qué hace durante el día? ¿Tiene trabajo?

10. ¿Usted está ganado dinero? ¿Trabaja fuera de su comunidad? ¿Dónde?

11. ¿Su pareja trabaja? ¿Qué es? ¿Él trabaja fuera de su comunidad? ¿Dónde?

*Preguntas*

1. ¿Usted entiende que es una enfermedad como la gripe? ¿Si, o no? ¿Qué es?
2. ¿De dónde ha recibido información sobre el sexo?
3. ¿Sabía usted lo que pasaba durante su primer menstruación? ¿Que paso?
4. ¿Sabe qué personas pueden contraer enfermedades por tener sexo?
5. ¿Sabe usted los efectos de una enfermedad transmitida sexualmente? ¿Sí o no? ¿Cuáles son?
6. ¿Usted sabe como se puede transmitir una enfermedad sexual? ¿Cómo?
7. ¿Cree que existen muchos casos de enfermedades transmitidas sexualmente en la comunidad, sí o no? ¿Porque?
8. ¿Quién cree que tiene más enfermedades transmitidas sexualmente, mujeres o hombres? ¿Por qué?
9. ¿Sabe usted cómo se puede prevenir la transmisión de una enfermedad sexual? Si o no, y ¿Cómo?
10. ¿Sabe usted que son condónes?
11. ¿Cree usted que los hombres quieren usar condones, sí o no? ¿Por qué?
12. ¿Cree usted que las mujeres piden a los hombres que usen el condón, sí o no? ¿Por qué?
13. ¿Cree usted que los hombres se molestarían/enfadarían si una mujer quiere usar un condón, sí o no? ¿Por qué?
14. ¿Cree usted que muchas personas usan condones en su comunidad, sí o no? ¿Por qué?
15. ¿Sabe usted cómo usar un condón, sí o no?
16. ¿Qué haría usted si no quiere más hijos?
17. ¿Conoce usted algún método anticonceptivo para planificar la familia?
18. ¿Habla/hablará usted con sus niños de relaciones sexuales, sí o no? ¿Y de qué hablan?
19. ¿Habla usted con sus amigas de relaciones sexuales, sí o no? ¿Y de qué hablan?
20. ¿Si una mujer tiene una enfermedad sexual, cree usted que ella le va a decir a sus amigas, sí o no? ¿Por qué? ¿A su pareja/esposo, sí o no? ¿Por qué? ¿Al doctor, sí o no? Por qué?
21. ¿Habla de relaciones sexuales con su esposo, sí o no? ¿De qué hablan?
22. ¿Cree usted que los hombres casados tienen muchos amantes, sí o no? ¿Por qué?
23. ¿Cree que es aceptable que un hombre casado tenga amantes, sí o no? Por qué?
24. ¿Cree usted que es aceptable que un hombre golpea a su pareja si ella tiene relaciones sexuales con otros hombres? ¿Por qué?
25. ¿Qué debería hacer una mujer si su pareja tiene relaciones sexuales con otra mujer? ¿Cree usted que es aceptable que una mujer golpea a su pareja si él tiene relaciones sexuales con otras mujeres? ¿Por qué?
26. Si el doctor le dice que usted tiene una enfermedad transmitida sexualmente, porque su esposo tenía relaciones con otra persona, pero su esposo le dice que no ha tenido relaciones sexuales con otra persona, ¿a quién le va a creer: el doctor o su esposo? ¿Por qué?
27. ¿Si usted tuviera dolor mientras que orina, qué haría? ¿Y por cuánto tiempo lo toleria?
28. ¿Si usted tuviera flujo anormal, que haría? ¿Y por cuánto tiempo lo toleria?
29. ¿Si usted tuviera dolor de útero, que haría? ¿Y por cuánto tiempo lo toleria?
30. ¿Si usted tuviera picazón en sus partes genitales, que haría? ¿Y por cuánto tiempo lo toleria?
31. ¿Si usted estuve sangrando de sus partes genitales, pero no es por su menstruación, que haría? ¿Y por cuánto tiempo lo toleria?
32. ¿Si usted tuviera molestias que haría para ayudarla o curarla?
33. ¿Si el doctor le diera medicina para ayudarla o curarla, ¿lo tomaría? ¿Sí o no? ¿Por qué? ¿Usaría otros métodos adicionalmente a la medicina del doctor?
34. ¿Si usted tuviera una enfermedad transmitida sexualmente por su esposo, quería que el doctor le diga? ¿Sí o no? ¿Por qué? ¿Qué haría?
35. ¿Prefiere un doctor o una doctora? ¿Por qué?
