# Supplementary material for: Assessment of Maya women’s knowledge, attitudes, and beliefs on sexually transmitted infections in Guatemala: a qualitative pilot study
Source: BMC Womens Health. 2020 Mar 21;20:58. doi: 10.1186/s12905-020-00925-7 (PMC7085160; doi:10.1186/s12905-020-00925-7)
Supplement: Supplementary file 3 — Additional file 3. Interview Questions in English [file 12905_2020_925_MOESM3_ESM.docx]

**Additional File 3. Interview Questions in English**

*Demographic Questions*

1. What sector do you live in?

2. What’s your full name?

3. How old are you?

4. Have you attended school? For how long?

5. How many kids do you have?

6. How many people live in your home? What is your relation to them?

7. Do you have a partner, boyfriend, or husband?

8. How old were you when you got married?

9. What do you do during the day? Do you work?

10. Do you make money? Do you work outside of the community? Where?

11. Does your partner work? Doing what? Does he work outside the community? Where?

*Questions*

1. Do you understand what illnesses like the flu are? Yes or no? What are they?
2. Where have you received information about sex?
3. Did you know what was happening during your first menstruation? What happened?
4. Do you know that people can get sick from having sexual relations?
5. Do you know the effects of a sexually transmitted infection (STI)? What are they?
6. Do you know how STIs are transmitted? How?
7. Do you think there are a lot of cases of STIs in your community? Why or why not?
8. Do you think men or women have more STIs? Why?
9. Do you know how to prevent the transmission of STIs? How?
10. Do you know what condoms are?
11. Do you think men want to use condoms? Why or why not?
12. Do you think women ask men to wear condoms? Why or why not?
13. Do you think men would get annoyed or angry if a woman asked them to use a condom? Why or why not?
14. Do you think a lot of people in your community use condoms? Why or why not?
15. Do you know how to use a condom?
16. What would you do if you didn’t want to get pregnant?
17. Do you know any contraceptives used for family planning?
18. Do you speak to your kids/do you plan to speak to your kids about sexual relations? What do you talk about?
19. Do you talk to your friends about sexual relations? What do you talk about?
20. If a woman has an STI, do you think she would tell her friends? Why or why not? Would she tell her partner/husband? Why or why not? Would she tell the doctor? Why or why not?
21. Do you talk about sexual relations with your husband? What do you talk about?
22. Do you think married men have extramarital sexual relations? Why or why not?
23. Do you think men’s infidelity is acceptable? Why or why not?
24. What should a man do if his wife has sexual relations with another man? Do you think it’s acceptable for a man to hit his wife if she has sexual relations with other men? Why or why not?
25. What should a woman do if her partner has sexual relations with another woman? Do you think it’s acceptable for a woman to hit her husband if he has sexual relations with other women? Why or why not?
26. If your doctor told you you contracted an STI because your husband has been unfaithful but your husband tells you he has not had extramarital sexual relations, would you believe the doctor or your husband? Why?
27. If you had pain while urinating, what would you do? How long would you tolerate it?
28. If you had abnormal discharge, what would you do? How long would you tolerate it?
29. If you had pain in your uterus, what would you do? How long would you tolerate it?
30. If your genitals were itchy, what would you do? How long would you tolerate it?
31. If you had irregular genital bleeding, what would you do? How long would you tolerate it?
32. If you had discomfort or pain, what would you do to treat or cure it?
33. If the doctor gave you medicine to treat or cure you, would you take it? Why or why not? Would you take any medicines in addition to what the doctor gave you?
34. If you contracted an STI from your husband, would you want the doctor to tell you? Why or why not? What would you do?
35. Do you have a preference for male or female doctors? Why or why not?
